# Supplementary material for: Tunable Wide-Field Illumination and Single-Molecule Photoswitching with a Single MEMS Mirror
Source: ACS Photonics. 2021 Aug 25;8(9):2728–36. doi: 10.1021/acsphotonics.1c00843 (PMC8447260; doi:10.1021/acsphotonics.1c00843)
Supplement: Supplementary file 1 — ph1c00843_si_001.pdf [file ph1c00843_si_001.pdf]

Tunable Wide-Field Illumination and Single-Molecule Photoswitching with a Single MEMS Mirror

Lucas Herdly,[†] Paul Janin,[‡] Ralf Bauer,[‡] and Sebastian van de Linde[*,†]

†*Department of Physics, SUPA, University of Strathclyde, Glasgow, Scotland, UK*

‡*Department of Electronic and Electrical Engineering, University of Strathclyde, Glasgow, Scotland, UK*

E-mail: s.vandelinde@strath.ac.uk

Supplementary Information

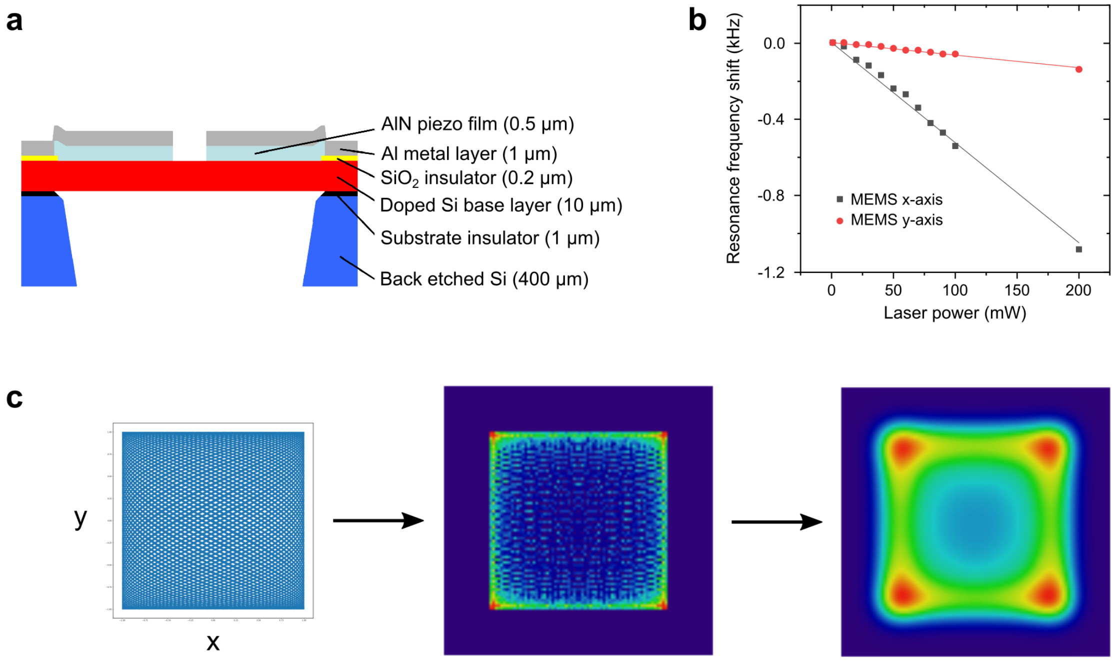

Figure S1: MEMS construction and functioning. **a**) Side view of the MEMS layer structure. **b**) We observed a linear shift of the resonance frequency with increasing applied laser power. As in our current prototype ~60% of the incoming light is absorbed, the material changed its mechanical properties and resonance frequencies due to an increase in temperature. **c**) Simulation of the micro-mirror movement; *Left*: A Lissajous pattern resulting of sinusoidal oscillations in x and y is transformed into a histogram (*Middle*) of the relative time spent in each point of the field of view (FOV) over a single cycle; *Right*: A convolution of the 2D histogram with a 2D Gaussian model of the laser beam produced the expected laser illumination of the camera FOV. The amplitude of the Lissajous pattern will change the resulting illumination (cf. Fig. S2).

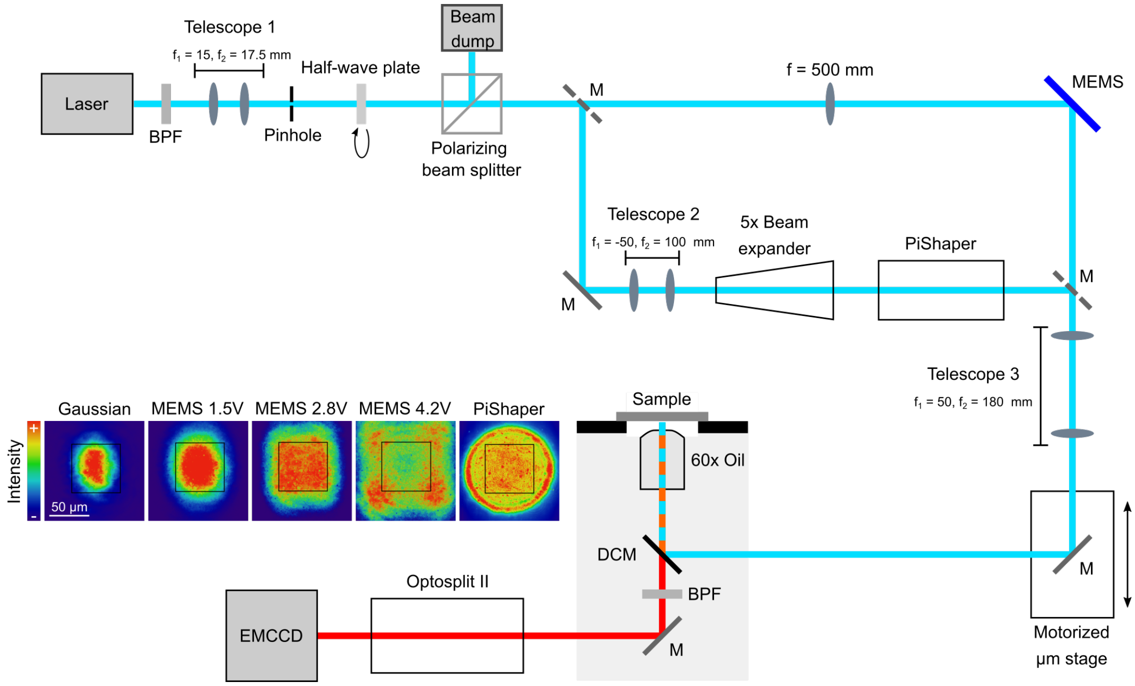

Figure S2: Schematic of SMLM setup. Lenses are indicated by focal length f; M indicates dielectric mirror, those with dashed gray lines are placed on magnetic holders that can be inserted or removed with high reproducibility; BFP bandpass filter, DCM dichroic mirror. Optical components for adjustments were left out for the sake of clarity. Fluorescence images of a µM concentrated ATTO655 solution in various illumination configurations are shown next to the detection path, where the central 512×512 px area is highlighted. Gaussian illumination was performed with the MEMS just used as conventional mirror.

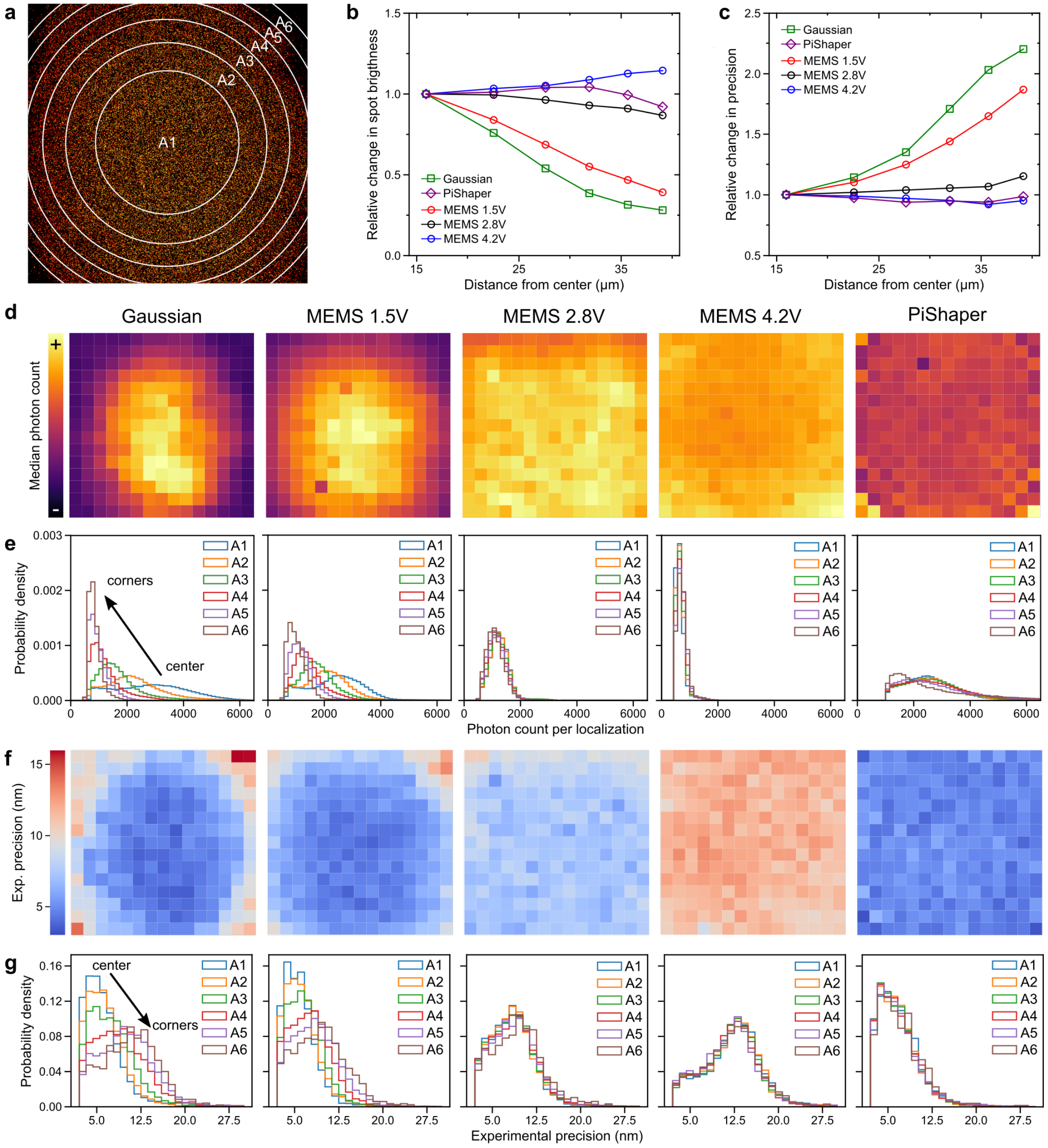

Figure S3: Spot brightness and localization precision. **a**) dSTORM image showing single-molecule localizations as well as ROIs for brightness and precision analysis: circular ROI (A1) and five annular ROIs (A2-6). **b**) The median photon count per localization normalized to the center value as function of the radius of ROIs A1-6; center photon counts were 2863, 2469, 1249, 629 and 2514 for Gaussian, MEMS 1.5 V, MEMS 2.8 V, MEMS 4.2 V and PiShaper, respectively. **c**) The experimental precision normalized to the center value as function of the radius of circular ROIs; center precision values were 4.36, 4.49, 7.24, 12.06 and 5.05 nm for Gaussian, MEMS 1.5 V, MEMS 2.8 V, MEMS 4.2 V and PiShaper, respectively. **d**) Photon count map; localizations were grouped in 225 ROIs, in which the median of the photon count per localization was determined for each ROI. **e**) Distributions of photon count for ROIs A1-6. Shift to low intensities for the MEMS 4.2 V can be assigned to the loss of laser power beyond the FOV. **f**) Map of the experimental localization precision. **g**) Distributions of precision for the ROIs A1-6. The shift to low precision for the MEMS 4.2 V is due to the loss of laser power beyond the FOV.

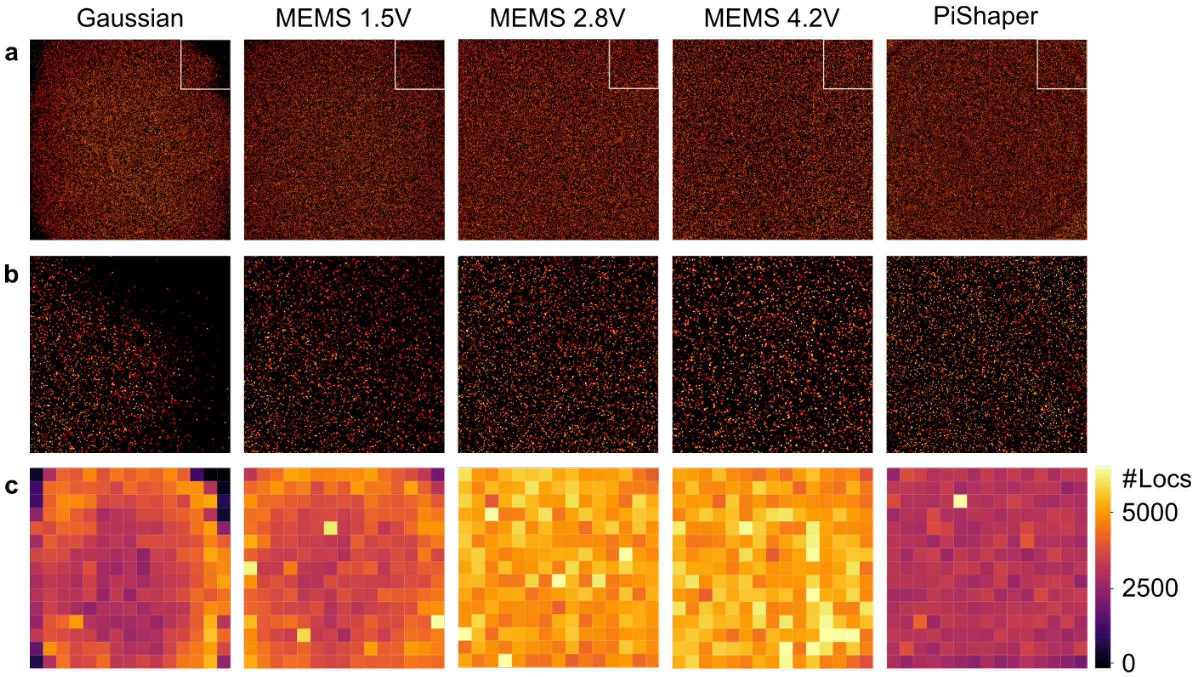

Figure S4: dSTORM images and localization density. **a**) dSTORM images (FOV 62.5 µm × 62.5 µm). **b**) Zoom in corner (white square in **a**); **c**) Localization count in each square region of interest. #Locs: number of localizations in each square ROI. It can be seen that in the corners many localizations were missed in Gaussian and MEMS 1.5 V illumination whereas MEMS 2.8 V, MEMS 4.2 V and PiShaper gave a homogeneous localization density over the FOV. The Gaussian illumination (and MEMS 1.5 V) showed a circular region of higher localization counts (**a**) compared to both the center and corners. In this region, the intermediate illumination power led to blinking events being observed over several consecutive frames with enough photons not to be discarded by the localization software. Due to high illumination power most blinking events in the center happened over the course of one or two frames, while the very low photon count in the corners led to the majority of events being missed or discarded.

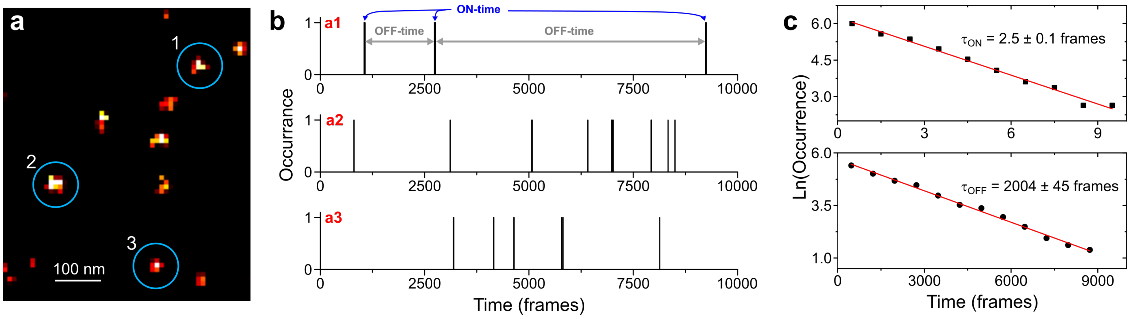

Figure S5: Analysis of experimental single-molecule time traces. **a**) Single localization patterns in the dSTORM image were subject to geometrical inspection. **b**) Their time traces were analysed and all ON- and OFF-times of all traces from the entire acquisition were summed into an ON-state and OFF-state histogram, respectively. **c**) The histograms were fitted to a single exponential decay using the function $\ln y = \ln a - kx$, with k as time constant and $1/k$ as the characteristic lifetime τ.

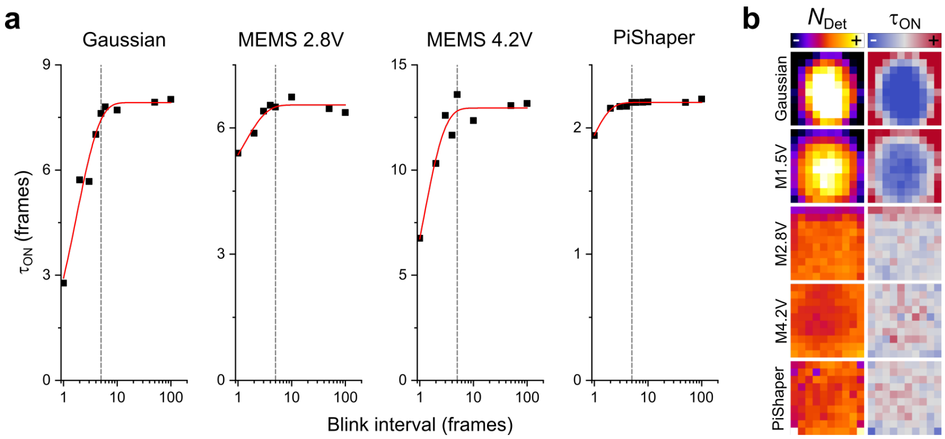

Figure S6: Determination of the ON-state lifetime τ_{on}. **a**) The effect of different blink intervals for consecutive localizations shown for different ROIs. A blink interval of 1 means that only localizations found in consecutive frames are assigned to the same ON-state; for example: a localization set found in frames 1, 2, 3, 4, 8, 9, 10 will create two ON-states of lengths 4 and 3 frames. With blink interval ≥ 4 only one ON-state is generated with a length of 10 frames. For all exemplary ROIs of the experimental data blink intervals > 5 (gray lines) did not further increase τ_{on}. Adapting the blink interval is important as in low intensity regions (e.g. Gaussian on the left) localizations will be missed by the localization algorithm. In high intensity regions (PiShaper, right) the effect is moderate but can still be observed. **b**) The resulting set of images: Spot brightness and τ_{on} maps as used for the analysis in Figs. 4&5. τ_{on} maps were generated using the proposed blink interval of 5 frames as indicated in **a**, which means that between two consecutive localizations of the same ON-state a gap of 4 frames was tolerated.

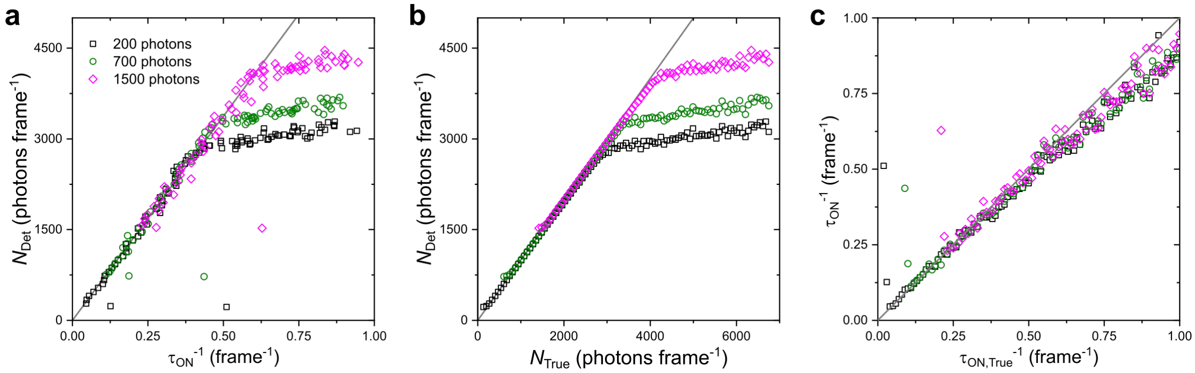

Figure S7: Kinetics analysis of a simulated data set for different photon thresholds. Key parameters were 0.1 s camera integration time, 340 nm spot size (fwhm) and linearly linked spot brightness N_{True} and ON-state lifetime $\tau_{\mathrm{on,True}}$ (see Methods). **a**) Detected spot brightness N_{Det} vs. τ_{on}^{-1} using the same analysis as for the experimental data as shown in Fig. 4; gray line indicates theoretical values (N_{True} vs. $\tau_{\mathrm{on,True}}$). **b**) Measured N_{Det} vs. simulated spot brightness (N_{True}), showing linear dependence up to ~3000 photons (threshold ≤ 700 photons). **c**) Measured τ_{on}^{-1} vs. simulated $\tau_{\mathrm{on,True}}^{-1}$, following the theoretical trend up to 0.6 frame^{-1}. Gray lines in **b** & **c** indicate the theoretical trend. Outliers in **a** & **c** can be assigned to underestimated lifetimes due to missed localizations. Photon thresholds of 200, 700 and 1500 photons are indicated by black squares, green circles and magenta diamonds, respectively.

Table S1: Photoswitching and resolution metrics. Statistics for data shown in **a**) Fig. 3 and **b**) Figs. 4&5. Bold values in **b** were used in Fig. 5. G = Gaussian illumination, M = MEMS, P = PiShaper. SD = standard deviation, MAD = median absolute deviation.

**a**)	τ_{off} (frames)			τ_{off}/τ_{on}			FRC resolution (nm)		
	G	M2.8V	P	G	M2.8V	P	G	M2.8V	P
Mean	2340	2477	2134	670	408	872	29.5	30.0	26.6
SD	498	171	181	260	45	83	5.4	2.3	1.7
SD/Mean	0.21	0.07	0.08	0.39	0.11	0.10	0.18	0.08	0.07
Min	1700	2048	1519	229	292	736	21.9	22.9	21.4
Max	4232	3078	2609	1262	551	1076	43.8	37.0	31.1
Median	2214	2470	2149	605	407	863	27.7	29.8	26.6
MAD	308	107	109	201	33	64	3.2	1.1	1.1

**b**)	N_{Det} (photons frame^{-1})					τ_{on} (frames)				
	G	M1.5V	M2.8V	M4.2V	P	G	M1.5V	M2.8V	M4.2V	P
Mean	1846	1796	1200	658	2571	4.35	4.30	6.13	12.50	2.46
SD	852	545	103	39	248	2.43	1.69	0.63	1.30	0.23
SD/Mean	**0.46**	**0.30**	**0.09**	**0.06**	**0.10**	**0.56**	**0.39**	**0.10**	**0.10**	**0.09**
Min	681	702	900	594	1913	1.48	2.25	4.99	9.30	1.45
Max	3489	2790	1351	802	3828	11.43	9.06	8.18	16.50	2.91
Median	1695	1792	1229	651	2560	3.68	3.90	5.98	12.51	2.48
MAD	740	452	42	26	113	1.66	1.06	0.41	0.75	0.13

Table S2: Comparison of different flat-field modes. [a] Without coatings, higher efficiency will be possible once metallic coatings are added; [b] different telescopes for different objectives; [c] can be reduced through image frame averaging; [d] inherent averaging.

	Gaussian Beam overfill[1]	Multimode fibre[2,3]	Micro-lenses[4]	PiShaper[5]	SLM[6]	ASTER[7]	This work
Price	0	$	$$	$$$	$$$	$$	$
Optical loss	High	50%	30%	Low	90%	Low	60%[a]
Additional Size	No addition	Medium	Medium	Medium	Large	Medium	Small
Field flatness	Non-uniform	Good	Good	Good	Good	Good	Good
Adaptability	Limited[b]	Medium	Medium	Limited	Good	Very Good	Very Good
Speckle	Yes	No	No	No	Yes[c]	No[d]	No[d]

References

(1) Wäldchen, S.; Lehmann, J.; Klein, T.; van de Linde, S.; Sauer, M. Light-induced cell damage in live-cell super-resolution microscopy. *Sci. Rep.* **2015**, *5*, 15348.

(2) Deschamps, J.; Rowald, A.; Ries, J. Efficient homogeneous illumination and optical sectioning for quantitative single-molecule localization microscopy. *Opt. Express* **2016**, *24*, 28080–28090.

(3) Zhao, Z.; Xin, B.; Li, L.; Huang, Z. L. High-power homogeneous illumination for super-resolution localization microscopy with large field-of-view. *Opt. Express* **2017**, *25*, 13382–13395.

(4) Douglass, K. M.; Sieben, C.; Archetti, A.; Lambert, A.; Manley, S. Super-resolution imaging of multiple cells by optimised flat-field epi-illumination. *Nat. Photonics* **2016**, *10*, 705–708.

(5) Stehr, F.; Stein, J.; Schueder, F.; Schwille, P.; Jungmann, R. Flat-top TIRF illumination boosts DNA-PAINT imaging and quantification. *Nat. Commun.* **2019**, *10*, 1268.

(6) Chen, S. Y.; Bestvater, F.; Schaufler, W.; Heintzmann, R.; Cremer, C. Patterned illumination single molecule localization microscopy (piSMLM): user defined blinking regions of interest. *Opt. Express* **2018**, *26*, 30009–30020.

(7) Mau, A.; Friedl, K.; Leterrier, C.; Bourg, N.; Lévêque-Fort, S. Fast widefield scan provides tunable and uniform illumination optimizing super-resolution microscopy on large fields. *Nat. Commun.* **2021**, *12*, 3077.
